# Gastric lipoma

**DOI:** 10.4103/0971-9261.70644

**Published:** 2010

**Authors:** M. Zameer, R. P. Kanojia, K. L. N. Rao, P. Menon, R. Samujh, B. R. Thapa

**Affiliations:** Department of Pediatric Surgery, PGIMER, Chandigarh, UT, India; 1 Department of Pediatric Gastroenterology, PGIMER, Chandigarh, UT, India

**Keywords:** Gastric lipoma, bleeding, childhood, gastric tumors

## Abstract

We report a 12-year-old-boy with gastric lipoma. Upper gastrointestinal (GI) endoscopy with biopsy and abdominal computed tomogram (CT) scan revealed the diagnosis. Open surgical excision of the mass with stomach preservation was done. The clinical presentation and management are discussed and the literature reviewed here. This is the sixth pediatric case reported in the English literature.

## INTRODUCTION

Benign gastric lipomas (GL) are rare in children; even in adult population, it represents only 2% of all gastric tumors.[1] They are characterized by the presence of a fatty tumor commonly located in submucosal layer of stomach. The tumor is mostly asymptomatic and indolent but may be symptomatic owing to its size and ulceration. Although several such cases have been documented in adults, we came across only five such pediatric reports of proven GL. We present a 12-year-old child with GL, who presented with hematemesis, malena and abdominal lump, along with a review of previous five pediatric patients reported in the literature.

## CASE REPORT

**Figure 1:**
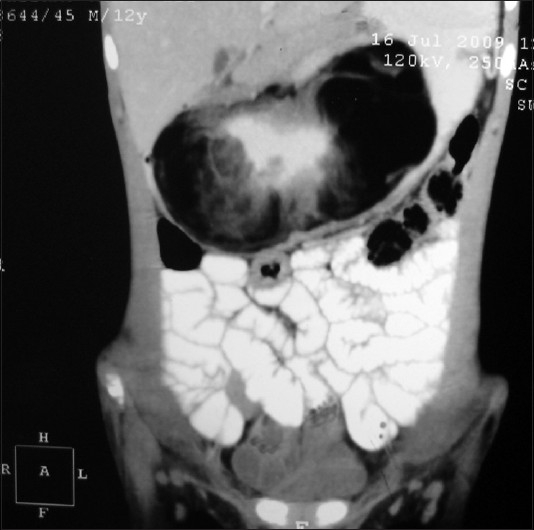
CT abdomen showing a large epigastric mass

**Figure 2:**
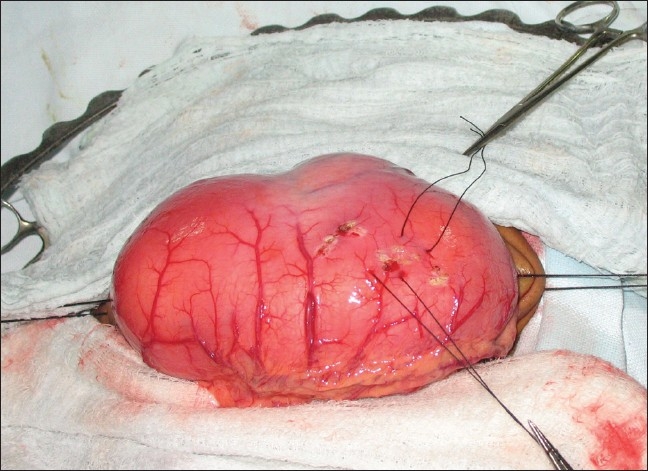
Tumor seen at surgery, arising from the intragastric submucosal area of antrum

A 12-year-old boy presented with the complaints of active hematemesis and malena of 1 week duration. He had a history of similar episode 6 months back during which he was treated with multiple blood transfusions. He also complained of a vague lump in the upper abdomen since 6 months. On examination, he was extremely pale with a firm mobile mass palpable in the upper abdomen. Laboratory investigations revealed hemoglobin of 3.6 g%. The coagulation profile was within the normal range. An ultrasound of the abdomen showed a large lobulated intraluminal mass in the stomach, following which a contrast-enhanced computed tomogram (CECT) scan of abdomen was done. CT showed a large ovoid intraluminal mass of fat attenuation with strands of soft tissue component involving the body and antral region of the stomach [Figure 1]. The possibility of the mass lesion being a lipoma, lipo-sarcoma or a teratoma was kept. An upper GI endoscopy showed a large sessile mass involving entire lesser curvature till incisura with a deep overlying mucosal ulcer. An endoscopic biopsy and the crush smear reported it to be lipoma with no evidence of malignant cells. After correction of the anemia, the child underwent laparotomy. On surgery, a large lipomatous mass measuring 12 × 8 cm occupying two-thirds of the stomach cavity was found. The mass was submucosal with an ulcer in the lesser curvature [Figure 2]. No serosal infiltration was seen. There was no evidence of perigastric or celiac lymph nodes. The tumor could be extirpated successfully by submucosal dissection along with a part of the gastric mucosa in the area where the ulcer was present. The postoperative course was uneventful. Histopathologic examination of the specimen confirmed the mass to be a benign submucosal lipoma. The child has completed 1 year of follow up and has not shown any evidence of recurrence in terms of symptoms or tumor.

## DISCUSSION

Gastrointestinal (GI) lipomas are tumors of mature adipose tissue, surrounded by a fibrous capsule.[2] GL constitute less than 1% of all the other gastric tumors, and of all the GI lipomas the chances of occurrence in gastric region is around 2%. The commonest location for a GI lipoma is colon.[3] The majority (95%) of GL originate from the submucosal layer, the remaining being either subserosal or intramural. The antrum is the most common location for these lesions.[4] The symptoms vary with the size of the tumor, with most tumors smaller than 4 cm being asymptomatic. The most frequent clinical manifestation is GI bleed (53%) which is due to ischemic ulceration of the overlying mucosa.[5] Other manifestations reported in literature are chronic anemia, abdominal pain, dyspepsia or obstruction.

Review of literature for GL reveled only five such cases in children. Stefan[6] reported a 12-year-old girl presenting with massive life-threatening hemorrhage, Macombe[7] reported a rare event of gastro-duodenal intussusception with a GL in child, Beck[5] reported a 13-year-old boy presenting with epigastric pain with hematemesis and palpable tumor. Endoscopic snare removal was first attempted on him, which was not successful. He was subsequently treated by open surgical removal. Alverti[8] reported an 11-year-old girl who was asymptomatic and the mass was detected incidentally; she was kept under observation with no treatment done. Antoniou[9] reported a 10-year-old boy with epigastric pain and malena, who underwent open surgical excision. The case discussed here is the sixth case in the literature, who presented with epigastric mass and pain and was treated by open surgical excision.

The radiological investigations and the endoscopic findings complement each other in the diagnosis of GL. CT is specific for lipomas located anywhere in the body; it shows a homogenous structure with a negative HU value, regular shape, no infiltrative growth, and sometimes a fibrous capsule. It permits a specific diagnosis on the basis of fat density of the tumor.[10] Magnetic resonance imaging (MRI) also aids in diagnosis, showing high signal intensity on T1 weighted sequences typical of a lipoma.[11] High resolution ultrasound has a detection rate of 93% in visualizing submucosal gastric masses.[12] GL reveal certain specific findings on upper GI endoscopy. The tenting sign, the naked fat sign, and the cushion sign are characteristic of GL.[13,14] Endoscopic biopsy confirms the histopathologic diagnosis of a lipoma.

Surgical resection is the mainstay of management of symptomatic large tumors. Endoscopic polypectomy has gained consensus on safety and efficacy for submucosal lesions smaller than 3 cm.[15] Larger sized tumors and broad based tumors have a higher risk of perforation. In adults, laparoscopic resection has been advised for lipomas less than 6 cm in diameter, though larger tumors have been excised laparoscopically.[16] Although malignant transformation has been reported in lipomas present in other parts of the body, GL are always benign and relapses do not occur.[17] In our case, a giant GL bled to cause severe life-threatening anemia. Prompt diagnosis and excision of the lesion was life saving.
